# The Crystal Structures of Yeast Get3 Suggest a Mechanism for Tail-Anchored Protein Membrane Insertion

**DOI:** 10.1371/journal.pone.0008061

**Published:** 2009-11-30

**Authors:** Junbin Hu, Jingzhi Li, Xinguo Qian, Vlad Denic, Bingdong Sha

**Affiliations:** 1 Department of Cell Biology, University of Alabama at Birmingham, Birmingham, Alabama, United States of America; 2 Department of Molecular and Cell Biology, Harvard University, Cambridge, Massachusetts, United States of America; University Paris 7, France

## Abstract

Tail-anchored (TA) proteins represent a unique class of membrane proteins that contain a single C-terminal transmembrane helix. The post-translational insertion of the yeast TA proteins into the ER membrane requires the Golgi ER trafficking (GET) complex which contains Get1, Get2 and Get3. Get3 is an ATPase that recognizes and binds the C-terminal transmembrane domain (TMD) of the TA proteins. We have determined the crystal structures of Get3 from two yeast species, *S. cerevisiae* and *D. hansenii*, respectively. These high resolution crystal structures show that Get3 contains a nucleotide-binding domain and a “finger” domain for binding the TA protein TMD. A large hydrophobic groove on the finger domain of *S. cerevisiae* Get3 structure might represent the binding site for TMD of TA proteins. A hydrophobic helix from a symmetry-related Get3 molecule sits in the TMD-binding groove and mimics the TA binding scenario. Interestingly, the crystal structures of the Get3 dimers from *S. cerevisiae* and *D. hansenii* exhibit distinct conformations. The *S. cerevisiae* Get3 dimer structure does not contain nucleotides and maintains an “open” conformation, while the *D. hansenii* Get3 dimer structure binds ADP and stays in a “closed” conformation. We propose that the conformational changes to switch the Get3 between the open and closed conformations may facilitate the membrane insertions for TA proteins.

## Introduction

Tail-anchored (TA) proteins represent a unique family of transmembrane proteins that contain a single transmembrane helix (about 25 residues) at the C-terminus. The N-terminal fragments of TA proteins are exposed to the cytosol. TA proteins can be found in the secretary pathways, nuclear envelope, peroxisomes and mitochondria. The TA proteins are present in all eukaryotic systems ranging from yeast to human [1]. It is estimated that human genome encodes more than 400 TA proteins [2]. A large number of the TA proteins target their C-terminal TMD into ER. The TA proteins localized at the ER membranes play central roles in protein secretion, folding, translocation and degradation [1], [3]. The TA proteins in mitochondria function to facilitate mitochondrial fission, protein translocation and apoptosis. Well-known TA protein examples include ER translocon member Sec61β, vesicle trafficking proteins SNAREs, the apoptosis-related protein Bcl-2 and signal transduction proteins such as PTP1B [2], [4], [5], [6], [7], [8], [9], [10].

The mechanisms how the TA proteins insert the TMD into membranes are distinct from the well-studied co-translational insertion pathway, which is mediated by the signal recognition particle (SRP), the ER-localized SRP receptor and the ER translocon formed by Sec61 complex [3], [11], [12]. Because TA proteins contain the TMD at the C-terminus, the main cytosolic fragment of the TA protein is delivered into the cytosol as it exits from the ribosome while the TMD remains in the ribosomal channel. This prevents the TA protein from utilizing the traditional co-translational membrane insertion pathways.

The TA protein insertion into ER membrane is ATP-dependent [6]. A soluble cytosolic ATPase TRC40/Asna-1 has been shown to interact with the newly synthesized TA protein Sec61β in the rabbit reticulocyte lysate (RRL) by biochemical studies [13]. The complex is then translocated to the ER membrane where the TMD of Sec61β is incorporated into ER membrane in an ATP-dependent fashion [13]. Disruption of TRC40 results in early embryonic lethality [14]. Recently yeast genetics and biochemical studies indicated that the post-translational insertion of the TA proteins into ER membrane requires the co-operation of the Golgi ER trafficking (GET) complex which contains Get1, Get2 and Get3 [15], [16], [17]. Get3 (also named as Arr4) is the yeast homologue of TRC40 of mammals. Get3 can recognize and bind the TMD of the TA proteins. Get1 and Get2 are ER transmembrane proteins which can recruit and form complexes with the TA protein-bound Get3. The GET complex carries out an energy-dependent process to facilitate the insertion of the TA protein TMD into the ER membrane. The complex formation of Get1, Get2 and Get3 ensures the specific TA protein insertion into the ER membranes. Loss of the GET complex results in the mis-localization of the TA proteins [15], [17]. New GET complex members such as Get4 and Get5 were identified that also play roles in TA protein membrane insertions [17].

The crystal structure of *E. coli* ATPase ArsA, which shares about 25% sequence identity with yeast Get3, is available [18]. Bacteria ArsA plays a role in metal detoxification, which is different from yeast Get3 or mammalian TRC40 [15], [19]. In this study, the crystal structures of Get3 from two yeast species, *S. cerevisiae* and *D. hansenii* suggest that Get3 may adopt two distinct conformations: an open conformation in nucleotide-free state and a closed conformation in ADP-bound state. A highly hydrophobic groove was identified as the binding site for the TMD of the TA proteins. We propose that the conformational changes of Get3 between the open and closed states may facilitate the membrane insertions for the TA proteins.

## Results and Discussion

### The *S. cerevisiae* Get3 Homo-Dimer Structure

**Figure 1:**
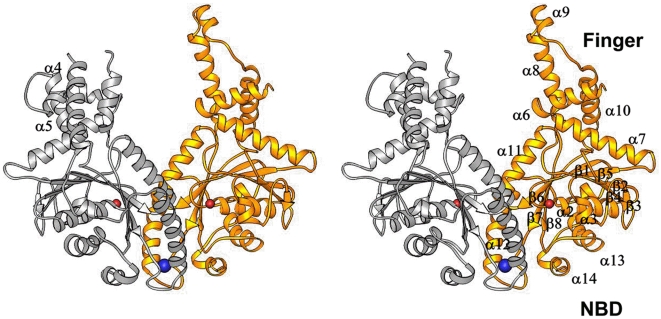
Ribbons drawing of the *S. cerevisia* Get3 homo-dimer structure in side-by-side stereo mode. The monomer A is in silver and the monomer B is in gold. The α-helices and β-strands are labeled in the structure. The nucleotide-binding domain (NBD) and the finger domain (Finger) are labeled. The Zinc ion is shown in a blue sphere and the two Mg ions at the nucleotide-binding sites are shown in red spheres. The missing parts of the structure are linked by dotted lines.

The crystal structure of *S. cerevisiae* full-length Get3 was determined to 2.3 Å resolution (Table 1). The resultant electron density map from the SAD phasing followed by two-fold molecular averaging was readily traceable. The Get3 forms a homo-dimer (molecules A and B) in the crystal structure (Fig. 1). In molecule A, residues 191 to 210 are missing and in molecule B, residues 93 to 116 are missing.

**Table 1:** Data collection, phasing and refinement statistics for *S. cerevisia* and *D. hansenii* Get3 structure.

	Native ScGet3	Se-Met ScGet3	DhGet3
**Data collection**			
Space group	*P*21212	P21212	I222
Cell dimensions			
*a*, *b*, *c* (Å)	218.53, 113.84, 48.22	219.40, 113.74, 48.37	59.85, 87.29, 230.59
		*Remote*	
Wavelength(Å)	0.9792	0.9750	0.9795
Resolution (Å)	2.3	3.1	1.8
*R*_sym_ or *R*_merge_	0.076(0.586)	0.102 (0.480)	0.039 (0.155)
*I*/sigma*I*	32.8(2.2)	22.7 (2.6)	42.9 (9.9)
Completeness (%)	99.3(93.7)	94.2 (59.7)	91.4(65.7)
Redundancy	6.2(4.3)	8.8 (4.4)	4.5(4.5)
**Refinement**			
Resolution (Å)	2.3		1.8
No. reflections	54950		49282
*R*_work_/*R*_free_	22.2(29.3)/24.5(34.8)		23.3(32.2)/24.7(38.5)
No. atoms			
Protein	5073		2082
Water	324		401
*B*-factors			
Protein	48.90		32.47
Water	53.52		39.70
R.m.s deviations			
Bond lengths (Å)	0.007		0.011
Bond angles (°)	1.1		1.3

*Highest-resolution shell is shown in parentheses.

The structure of the *S. cerevisiae* Get3 monomer consists of 14 α-helices (α1–α14) and eight β-strands (β1–β8) (Fig. 1). The Get3 structure contains a nucleotide-binding domain (NBD) and a smaller finger domain. The β-strands β 1 to β 8 form a large β-sheet which constitutes the core of the NBD. All the β-strands are parallel to each other except β3 which is anti-parallel to others. A1-A3 and A13-A14 are located on one side of the β-sheet and α11–α12 are on the other side of the sheet. This topology represents a Rossmann fold that is found in many nucleotide-binding domains. One Magnesium ion can be located at the ATP-binding site in the NBD but no nucleotide molecules can be found even in the presence of ATP (or ADP) in the crystallization conditions. The small finger domain of Get3 protrudes out of the ATPase domain. The finger domain of Get3 consists of helices α4 to α10. The finger domains appear to be flexible in the crystal structure because helices α8 and α9 are missing in monomer A. In the finger domain of monomer B, helix α4 is missing.

**Figure 2:**
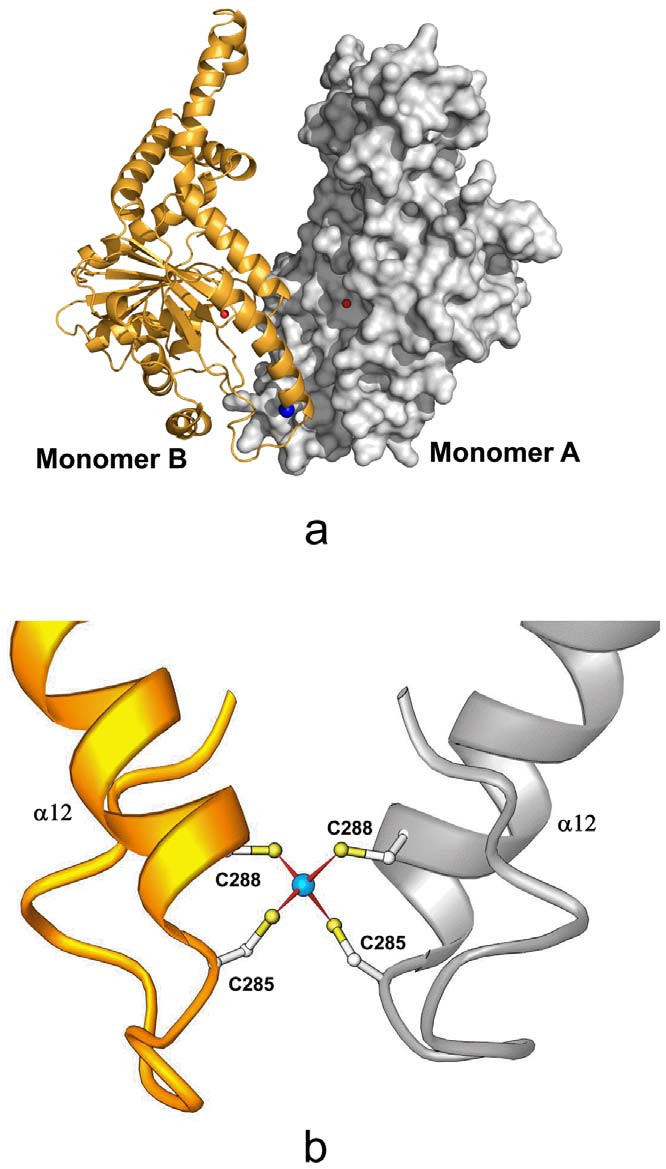
Dimerization of *S. cerevisia* Get3 molecule. a) The Get3 dimer illustrated by PYMOL. The molecular surface of the Monomer A is shown in silver and Ribbons drawing of the monomer B is shown in gold. The orientation of this Get3 dimer is about 180° rotation along the vertical axis from that in Fig. 1. The dimer interface is located at the bottom of the structure. The Zinc ion is shown in a blue sphere and the two Mg ions at the nucleotide-binding sites are shown in red spheres. b) The inter-molecular Zinc-finger motif. The Zinc ion is bound by C285 and C288 from the N-terminal end of helix A12 from monomer A and B, respectively. C285, C288 and α12 are labeled. The Zn ion is shown in a blue sphere.

**Figure 3:**
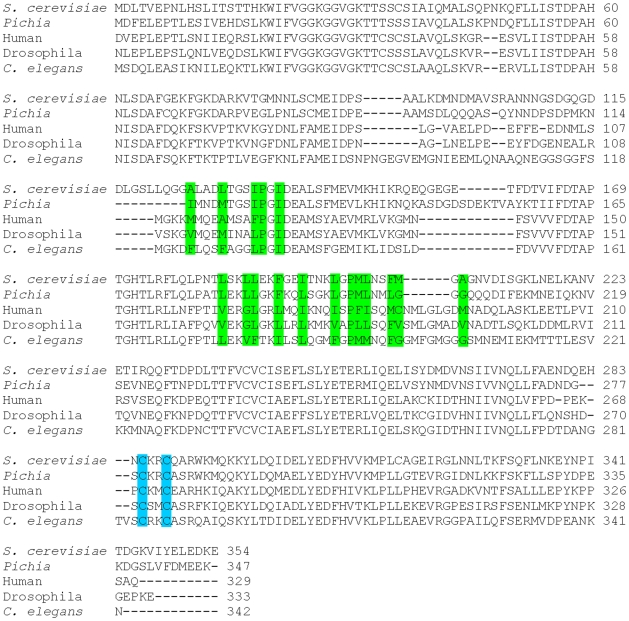
Sequence alignment of the Get3 family members. Program ClustalW was utilized to align the Get3 sequences from *S. cerevisiae* (yeast) with those from *D. hansenii, H. sapiens*, *D. Melanogaster* and *C. elegans*. The amino acid residues are numbered at the right side of the alignment. The conserved C285 and C288 responsible for the Zinc-finger motif formation are labeled in blue. The conserved hydrophobic residues involved in forming the TMD-binding groove are labeled in green.

In the asymmetric unit, two Get3 monomers form a dimer through a non-crystallographic two-fold axis. The dimer interface of Get3 is about 1100 Å^2^, which accounts for only 6% of the monomer surface area. The relatively small dimer interface between the two Get3 monomers indicates possible flexibility between them. The two Get3 monomers are associated together primarily through a novel inter-molecular Zinc-finger motif, which was not previously revealed by Get3 sequence (Fig. 2a, b). In the crystal structure, one Zinc ion is coordinated by four Cys residues, C285 and C288 from molecule A and B, respectively. C285 and C288 protrude out from the helix α12 in the structure and form the Zinc finger motif with the C285 and C288 from another monomer. This inter-molecular Zinc finger motif provides the major driving force for Get3 dimerization since no other major contact areas can be identified from the dimer interface. Sequence alignment shows that C285 and C288 are absolutely conserved among the Get3 family members from yeast to human (Fig. 3).

### The Transmembrane Domain (TMD)-Binding Groove of the *S. cerevisiae* Get3

**Figure 4:**
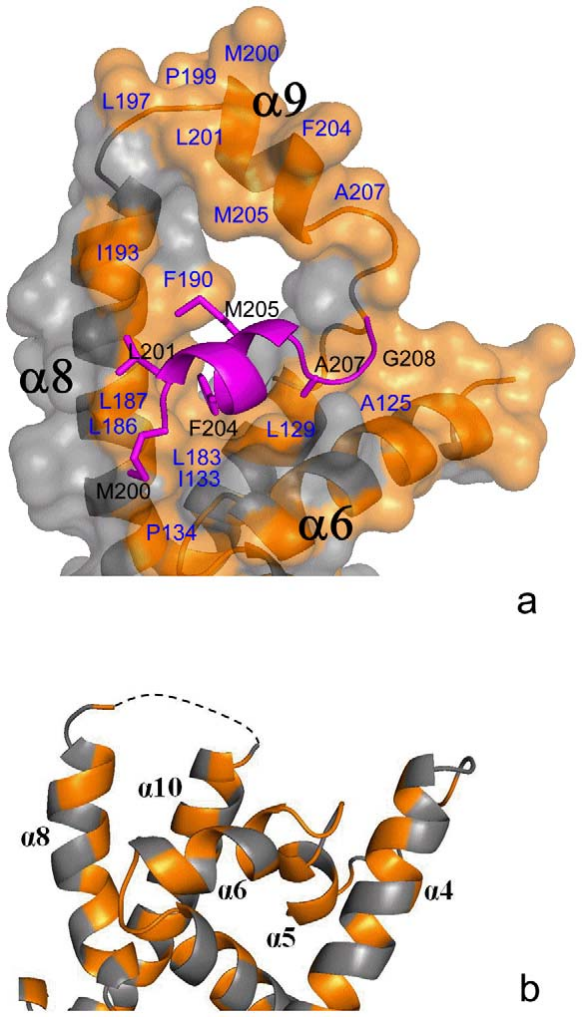
The TMD-binding groove of *S. cerevisia* Get3. a) The hydrophobic surface of Get3 molecule is shown in gold. The orientation of the finger domain is similar as that in Fig. 1. The hydrophobic residues constituting the TMD-binding groove are labeled in blue. The extended helix α6, α8 and α9 in the finger domain of monomer B are labeled. Helix α9 from the symmetry-related Get3 molecule is shown in magenta. The hydrophobic residues from helix α9 are labeled in black. b) The finger domain of Get3 in the monomer A. This finger domain is positioned to the similar orientation as for that in Fig. 1b. The hydrophobic residues are shown in gold. The missing part of the structure is shown in dotted line. Helices α4, α5, α6, α8 and α10 are labeled. The helices α5 and α6 in this figure are merged into an extended helix α6 in Fig. 4a.

Biochemical and genetics studies have shown that Get3 directly interacts with the TMD of the TA proteins [15], [16], [17]. Because of the highly hydrophobic nature of the TMD of TA proteins, we examined the hydrophobic surfaces of Get3 molecule to identify a potential TMD-binding site. A large hydrophobic groove is found on the finger domain of Get3 structure that might represent the binding site for TMD of TA proteins (Fig. 4a). One side of this groove is formed by helix α6. The other side of the groove is generated by helices α8 and α9. This hydrophobic surface of this groove is constituted primarily by residues A125, L129, I133 and P134 from helix α6, residues L183, L186, L187, F190, I193 and L197 from α8 and residues P199, M200, L201, F204, M205 and A207 from α9 (Fig. 4a). This groove represents the largest hydrophobic surface of the Get3 molecule. The hydrophobicity of residues forming the groove is nicely conserved among the Get3 family members (Fig. 3), suggesting that this hydrophobic groove is a common feature for Get3 from difference species.

Strong support that the hydrophobic groove on the Get3 finger domain can accommodate the TMD from TA proteins comes from the crystal packing. In monomer B of the Get3 dimer, a hydrophobic helix α9 from a symmetry-related Get3 monomer makes strong hydrophobic interactions with the putative TMD-binding groove (Fig. 4a). This hydrophobic helix α9 sits anti-parallel to the helix α6 which forms one side of the groove. The hydrophobic side chains from residues M200, L201, F204, and M205 from helix α9 make extensive interactions with hydrophobic residues from residues L186, L187, F190 and I193 from helix α8 at the TMD-binding groove. We propose that this helix α9 from symmetry-related monomers may mimic the binding status for TMD of TA proteins for Get3. The hydrophobic helix α8 may also play an important role in stabilizing the TMD of TA protein in the TMD-binding groove by acting as a “finger”.

In monomer A of the Get3 dimer, no helix is present in the TMD-binding groove due to different crystal contacts. It is tempting to postulate that monomer A may exhibit the ligand-free state and the monomer B may represent the ligand-bound state for Get3 (Fig. 4a, b). It is interesting to note that one side of the groove (helices α8 and α9) is disordered in monomer A while it is ordered in the monomer B's electron density map. This observation indicates that TMD binding may stabilize the Get3 TMD-binding groove which would otherwise be flexible when empty. Moreover, in monomer B, the helix α5 merges with helix α6 and forms an extended helix α6 while in monomer A, helix α5 and helix α6 are positioned at an angle of about 90°. Thus, the TMD binding may promote the merging of the helices α5 and α6, resulting in the extension of the binding site for the TMD.

Very recent structural studies on yeast Get3 indicated that the long helix α7 may be also involved in the TA protein binding [20], [21]. In the published Get3 structures (PDB code 2WOO, 3.0 Å resolution; PDB code 3IDQ, 3.7 Å resolution), a major part of the finger domain that includes helices α8 and α9 is missing. Therefore, it is likely that Get3 may adopt multiple conformations to interact with the TMD of TA proteins.

### The *D. hansenii* Get3 Homo-Dimer Structure

**Figure 5:**
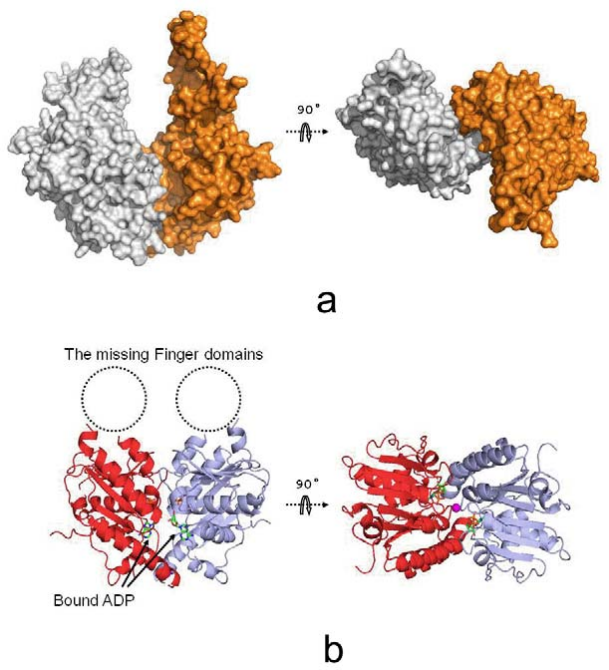
The structural comparison between *S. cerevisia* and *D. hansenii* Get3 dimer. a) The molecular surface drawing of the Get3 dimer by Pymol. The monomer A is in silver and the monomer B is in gold. The orientation of the Get3 dimer in the left panel is similar as that in Fig. 1. b) The Ribbons drawing for *D. hansenii* Get3 homo-dimer. One monomer is in red and the other is in blue. The missing finger domains are indicated in dotted circle. The Zinc ion is shown in a pink sphere. The bound ADP molecules are labeled and shown in rod model.

The *S. cerevisiae* Get3 crystal structure was determined free of nucleotides. We were not able to grow crystals of *S. cerevisiae* Get3 complexed with nucleotide. To obtain the nucleotide-bound Get3 crystal structure, we have expressed and purified Get3 from another yeast species, *D. hansenii.* We were able to crystallize *D. hansenii* Get3 complexed with ADP. The crystal structure of *D. hansenii* Get3 was determined to 1.8 Å resolution using molecular replacement method (Table 1). The *D. hansenii* Get3 forms a homo-dimer in the high resolution crystal structure (Fig. 5b). The bound ADP molecule can be clearly identified in the Get3 nucleotide-binding domain (NBD) in the crystal structure. The finger domain in the *D. hansenii* Get3 structure, however, is missing in the electron density map (Fig. 5b).

The *D. hansenii* Get3 shares 65% sequence identity with the *S. cerevisiae* Get3. When we compare the *S. cerevisiae* Get3 homo-dimer structure with the *D. hansenii* Get3, a striking feature is that the two Get3 monomers are associated in a very distinct fashion. In the *S. cerevisiae* Get3, the two Get3 monomers are swung away from each other. In the *D. hansenii* Get3 structure, the two nucleotide-binding domains (NBD) face to each other and make close contacts. The distance of the two Mg ions located in the NBDs within *S. cerevisiae* Get3 dimer is 26 Å while the two Mg ions located in the NBDs of *D. hansenii* Get3 dimer are spaced only ∼5 Å apart. In the *S. cerevisiae* Get3 dimer structure, the two monomers are twisted away from each other by 36° from the positions of their counterparts in the *D. hansenii* Get3 homo-dimer structure (Fig. 5a, Fig. 5b).

The *S. cerevisiae* Get3 structure was solved free of nucleotides and the *D. hansenii* Get3 structure was complexed with ADP. We hypothesize that Get3 may adopt an “open” conformation in nucleotide-free state and a “closed” conformation in ADP-bound state. The *S. cerevisiae* Get3 crystal structure represents the open conformation while the *D. hansenii* Get3 structure exhibits the closed conformation. In the *D. hansenii* Get3 structure, the bound ADP plays an important role in associating the two Get3 monomers in the closed conformation. The flexibility between the two Get3 monomers may provide evidences that the conformational changes to switch Get3 between the open and closed states may be possible.

The recently published Get3 structures from *S. cerevisiae* and *S. pombe* indicated that Get3 may exhibit open conformation in nucleotide-free state (PDB code 2WOO; resolution 3.0 Å) and closed conformation in ADP-ALF4-bound state (PDB code 2WOJ; resolution 2.0 Å), which is consistent with our findings [21]. The closed Get3 structure (2WOJ) showed an extended hydrophobic groove possibly for TA protein binding in the finger domain while in the open conformation (2WOO), the hydrophobic surfaces of the groove are shielded. In an independent study, both Get3 structures from *A. fumigatus* complexed with ADP (PDB code 3IBG; resolution 3.2 Å) and Get3 from *S. cerevisiae* in nucleotide-free state (PDB code 3IDQ; resolution 3.7 Å) were determined in the open conformations [20]. However, in this paper the structure of *D. hansenii* Get3 complexed with ADP was found in closed conformation. It is possible that Get3 in the ADP-bound state may exist in both open and closed conformations, which might be important for its biological functions. In the Get3 structure from *A. fumigatus* complexed with ADP (3IBG), three homo-dimers are associated into a hexamer through the finger domain interactions. The finger domains appear to be quite flexible in the Get3 structure because all the Get3 structures do not contain the intact finger domain in the electron density map. In some cases, the finger domains are completely missing possibly due to lack of crystal contacts. The flexibility of the finger domain may render Get3 the ability to accommodate the TMD of TA proteins with different sequences.

### The Working Model for Get3 to Facilitate TA Protein Biogenesis

**Figure 6:**
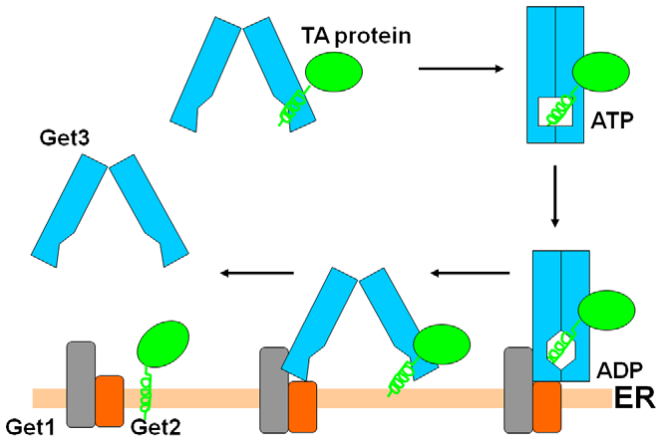
The working model for Get3 to facilitate the TA protein biogenesis. The TMD of the TA protein is shown in a green helix. Get1, Get2 and Get3 are labeled. The ATP-bound state and ADP-bound state of Get3 are labeled.

We have proposed a working model how Get3 facilitates the TA protein biogenesis by switching between open and closed conformations (Fig. 6). In the open conformation, the two TMD-binding grooves within one Get3 dimer can interact with the TMD of the nascent TA proteins to initiate the TA protein membrane insertions. The ATP binding of Get3 may switch Get3 to the closed conformation, where the two TMD-binding grooves from the two Get3 monomers may approach each other to form an enclosed binding site to protect the TMD of the TA proteins. The TA protein-bound Get3 docks with the ER-located Get1-Get2 complex. The Get1-Get2-Get3 complex formation may catalyze the ADP release from Get3 that will switch Get3 to the open conformation to expose the TMD of TA proteins for membrane insertions.

We have modeled the Get3 dimer in the closed conformation based on the *D. hansenii* Get3 dimer. In the modeled structure, the finger domains from two Get3 monomers collide with each other (data not shown), suggesting that the Get3 finger domains may undergo conformational changes when the Get3 dimer switches from the open to the closed conformation.

## Materials and Methods

### Structure Determination of *S. cerevisia* Get3

The protein expression and crystallization of the yeast Get3 has been previously described [22]. Briefly, the recombinant proteins of Get3 were expressed from *E. coli* and purified using Ni-chelating column and gel-filtration column. The Get3 proteins were crystallized by hanging drop vapor diffusion method with the well solution of 100 mM MES buffer (pH 6.0), PEG 3350 15%, 0.2 M Ammonium Sulfate.

The native Get3 crystals diffracted X-ray to 2.3 Å at beamline GM-CAT in APS. We solved the crystal structure of Get3 by using Se-Met Get3 crystals through the single-wavelength anomalous dispersion (SAD) method (Table 1). The SAD data set for Se-Met Get3 crystals were collected at SER-CAT in APS. The Selenium positions were located by use of the program ShelxD and the initial phasing was carried out by using SOLVE [23]. The subsequent phase improvement was performed by using RESOLVE [23].

The resultant electron density map was traced using program COOT [24]. Refinement was carried out using the program Refmac5 [25]. The native data set was used for the refinement. (Table 1).

### Structure Determination of *D. hansenii* Get3

The crystal structure of *D. hansenii* Get3 was crystallized by hanging drop vapor diffusion method with the well solution of 0.2M ammonium mono-basic phosphate, 20% Glycerol, 1 mM MgCl_2_, 5 mM KCl and 2 mM ADP. The data collection was carried out at beamline 7–1 at SSRL. The *D. hansenii* Get3 crystal structure was determined by using molecular replacement method using the Get3 monomer as the search model. Program Phaser was utilized to carry out the molecular replacement method [25].

### Accession Number

Atomic coordinates of *S. cerevisia* and *D. hansenii* Get3 have been deposited in the Protein Data Bank under the accession codes of 3H84 and 3IO3.
